# Photothermal 2D Nanosheets Combined With Astragaloside IV for Antibacterial Properties and Promoting Angiogenesis to Treat Infected Wounds

**DOI:** 10.3389/fbioe.2021.826011

**Published:** 2022-02-09

**Authors:** Lichang Liu, Wenfeng Wang, Weihong Hong, Yuyan Jin, Lichun Wang, Sujun Liu, Ailin Wang, Xusheng Liu

**Affiliations:** ^1^ Department of Nephrology, Zhuhai Hospital of Guangdong Provincial Hospital of Chinese Medicine, Zhuhai, China; ^2^ Second Clinical Medical College, Guangzhou University of Chinese Medicine, Guangzhou, China; ^3^ Department of Nephrology, Guangdong Provincial Hospital of Chinese Medicine, Guangzhou, China

**Keywords:** infected wound healing, anti-inflammatory, photothermal therapy, Astragaloside IV (AS), black phosphorus (BP) nanosheets

## Abstract

Bacterial infection, inflammatory disorder, and poor angiogenesis of tissue in chronic wounds are the main reasons why wounds are difficult to heal. In this study, a novel MSN-PEG@AS/BP nano-spray was designed to solve these issues. Astragaloside IV (AS) was loaded in mesoporous silica nanoparticles (MSN) to enhance angiogenesis and regulate inflammation, and the two-dimensional (2D) nanosheet black phosphorus (BP) was used to kill bacteria through a photothermal effect. Under thermal decomposition, the covalent bond of polyethylene glycol (PEG) was broken, releasing AS to promote the proliferation of fibroblasts, the formation of blood vessels, and the resolution of inflammation. AS can promote the polarization of the anti-inflammatory (M2) macrophage phenotype to enhance the deposition of extracellular matrix and the formation of blood vessels. Besides, BP showed a significant photothermal effect and nearly 99.58% of *Escherichia coli* and 99.13% of *Staphylococcus aureus* were killed in an antibacterial study. This nano-spray would be a novel therapeutic agent for infected wound treatment.

## 1 Introduction

The skin is the largest organ of the human body. When the skin is severely damaged, it will seriously threaten human health (Moreira and Marques, 2022). In fact, chronic wounds are currently facing a huge challenge in the global healthcare system. Despite a series of treatments for wounds in clinic, there are still some hard-to-heal wounds (Clark and Price, 2004). The annual cost for treating chronic wounds in the United States exceeds 25 billion U.S. dollars. Some chronic wounds that are difficult to heal, such as diabetic foot ulcers and venous ulcers of the lower extremities, may lead to infection, sepsis, amputation, and even death (Sen et al., 2009). Therefore, wound dressings that can effectively treat chronic wounds are urgently needed.

Chronic wounds are often accompanied with persistent infection, excessive inflammation, and damaged tissue (Cavanagh et al., 2005). Bacterial infection is a major factor hindering the healing of chronic wounds. The biofilm formed by bacterial infection not only destroys the host’s immune system, but also forms a barrier that hinders the tight junction of epithelial cells, resulting in chronic wounds that cannot be closed (Metcalf and Bowler, 2013). Traditional treatment method is to use a lot of antibiotics on the infected wounds. However, bacterial resistance may occur during treatment. In order to avoid the long-term use of antibiotics that leads to bacterial resistance, photothermal therapy (PTT) was chosen as new way to treat the infected wounds. Due to its non-invasiveness, high-efficiency bactericidal properties, and low toxicity, PTT has attracted widespread attention in recent years (Chen et al., 2020). The emergence of black phosphorus (BP) nanosheets provides a new method to solve the problem of bacterial resistance. BP has an excellent photothermal conversion effect that can destroy bacterial biofilms under NIR irradiation (Miao et al., 2019). In addition, the large surface area and planar structure of BP provide convenience for loading wound healing drugs (Wang et al., 2015). Moreover, BP has excellent biocompatibility and biodegradability, and its degradation products in aqueous media are non-toxic phosphate and phosphonate (Huang et al., 2018).

Excessive inflammation is another major problem in wound healing, which leads to growth factor and ECM degradation, and hinders the formation of new granulation tissue and blood vessels (Eming et al., 2014). Macrophages play an essential role in the resolution of inflammation. The conversion of the M1 phenotype of macrophages to the M2 phenotype is conducive to changing the wound microenvironment from a pro-inflammatory state to an anti-inflammatory state (Larouche et al., 2018). Previous studies have shown that a certain concentration of silicon ions can promote the polarization of macrophages to the M2 phenotype (Zhu et al., 2020). Nano-silica is widely used as drug carriers, and the silicon ions produced during the degradation process can control inflammation. Meanwhile, nano-silica can also be used as a carrier to release other drugs. Mesoporous silica nanoparticles (MSN) are newly derived by a template etching method (Nguyen et al., 2020). Compared to nano-silica, MSN effectively solves the problem of drug loading efficiency and sustained release kinetics due to its high surface area and ordered mesoporous channels (Zhang et al., 2017).

Astragaloside IV (AS), the main component of Astragalus, has been demonstrated to be the most effective biologically active compound to promote skin cell proliferation (Lee et al., 2018). As reported in the literature, AS regulates nitric oxide (NO) and growth factors through the JAK2/STAT3 pathway to induce angiogenesis in umbilical vein endothelial cells (Wang et al., 2013). In addition, AS could downregulate the pro-inflammatory cytokines and upregulate anti-inflammatory cytokines, such as transforming growth factor-β (TGF-β), in the inflammation site, and trigger the transition of the macrophage phenotype to the anti-inflammatory state (M2) (Chen et al., 2012). Anti-inflammatory macrophages contribute to the production of extracellular matrix (ECM) and wound contraction. Meanwhile, M2 macrophages guide the tunnel of endothelial migration and release angiogenic factors, thereby promoting angiogenesis. Chen et al. have confirmed that AS can promote wound re-epithelialization, angiogenesis, and ECM remodeling (Chen et al., 2012).

**SCHEME 1:**
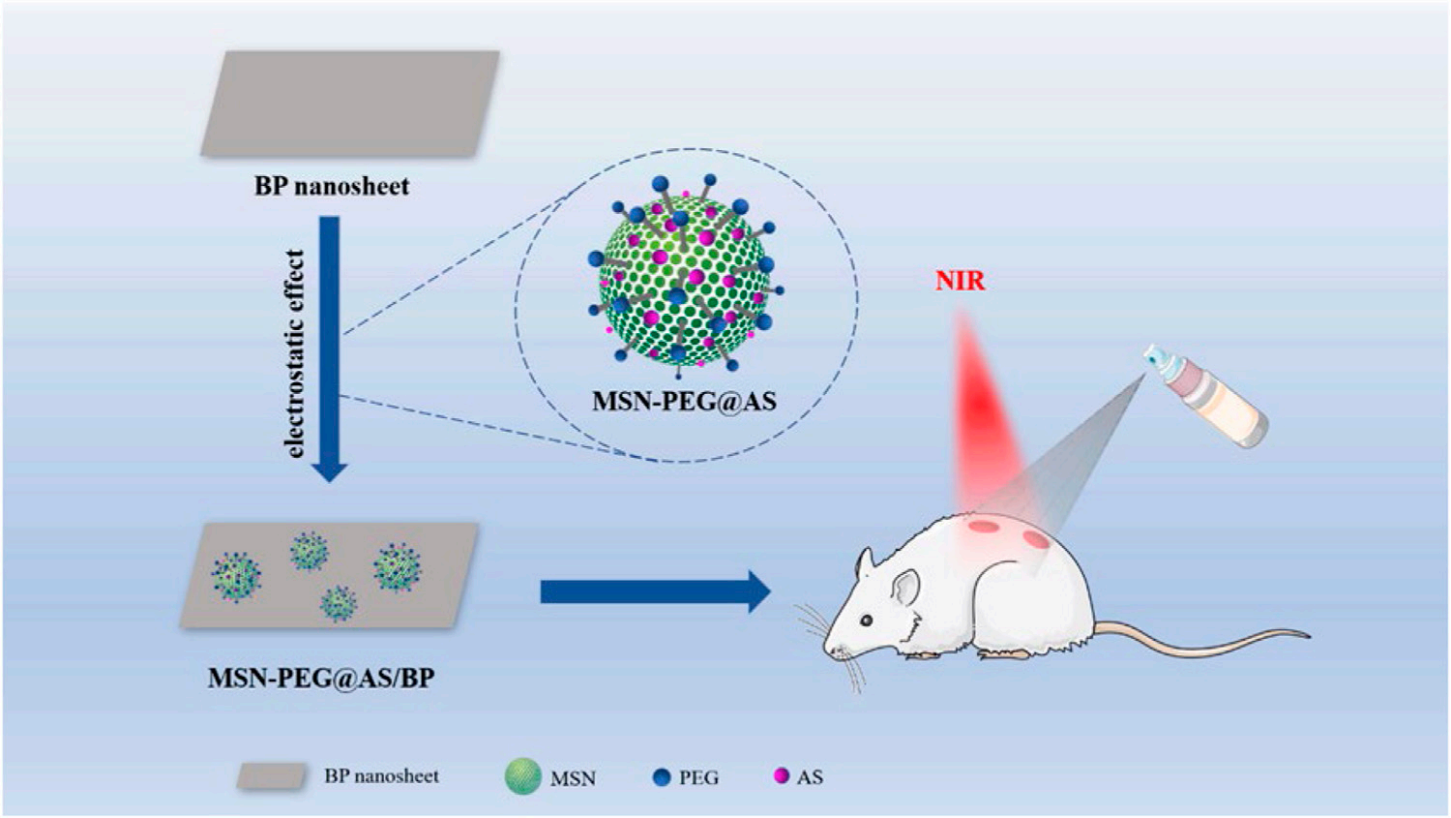
Schematic illustration of the construction of the MSN-PEG@AS/BP multifunctional nano-spray for bacteria-infected wound, enhancing angiogenesis and regulating inflammation.

In this research, we loaded AS in the mesopores of aminated MSN (MSN-NH_2_), and complex with BP through electrostatic adsorption to produce MSN-PEG@AS/BP nano-spray. The photothermal effect of this nano-spray irradiated by NIR laser can generate local high temperature to destroy the structure and metabolism of bacteria, thereby killing them. Considering that the photothermal effect of BP caused physical changes (phase transition, swelling, and solubility) in MSN, which limited the release rate of AS, we designed a thermally responsive PEG shell grafted on the MSN surface loaded with AS and BP. When PEG is thermally decomposed, the covalent bond is broken to trigger the release of the drug (Saint-Cricq et al., 2015). The nano-spray reported here is a new way to treat bacteria-infected wound (Scheme 1).

## 2 Experimental Section

### 2.1 Materials

Cetyl trimethyl ammonium chloride (CTAC), ethyl orthosilicate (TEOS), 3-aminopropyltriethoxysilane (APTES), p-toluenesulfonyl chloride, p-hydroxybenzaldehyde, Astragaloside IV (AS), anhydrous magnesium sulfate (MgSO_4_), dichloromethane (CH_2_Cl_2_), ethanol, methylbenzene, and methanol were purchased from Macklin (Shanghai, China). Triethanolamine (TEA) and polyethylene glycol (PEG) were purchased from Aladdin Industrial Corporation (Shanghai, China). Hydrochloric acid (HCl) and sodium chloride (NaCl) were purchased from Sinopharm Chemical Reagent Co., Ltd. (Shanghai, China). BP crystal was purchased from Nanjing XFNANO Materials Tech. Co. Ltd. (Nanjing, China). All other reagents used in the experiment were of analytical grade and no further purification was required.

### 2.2 Synthesis of MSN-NH_2_

MSN was synthesized by a template etching method (Wu et al., 2018). Briefly, CTAC (2 g) and TEA (0.1 g) were dissolved in 20 ml of deionized water at 95°C for 1 h under stirring. Then, 1.5 ml of TEOS was added in the above solution. After stirring for 1 h, the mixture was centrifuged at 15,000 *g* for 15 min and washed three times with ethanol to obtain the MSN.

For amination modification, the product was dispersed in 50 ml of ethanol. Two hundred microliters of APTES was added to the solution and refluxed for 4 h at 60°C under stirring. The mixture was then centrifuged and washed with deionized water to obtain the aminated mesoporous silica (MSN-NH_2_). The final product MSN-NH_2_ was dispersed in ethanol and stored at 4°C.

### 2.3 Synthesis of Dibenzaldehyde-Terminated Polyethylene Glycol (OHC-PEG-CHO)

OHC-PEG-CHO was prepared by a two-step process (Yang et al., 2017). Polyethylene glycol (16 g, 4 mmol) was added to azeotropically dry until most of the toluene was distilled, and then anhydrous CH_2_Cl_2_ (50 ml) and triethanolamine (2.22 ml, 16 mmol) were added to it. After cooling to 0°C, p-toluenesulfonyl chloride solution (1.24 ml, 60 mM, dissolved in CH_2_Cl_2_) was added dropwise to the above mixture during 30 min and stirred for 24 h at room temperature, and then 100 ml of deionized water was added and the mixture was extracted five times with 25 ml of CH_2_Cl_2_. The organic layer was washed by 1 M HCl and saturated NaCl solution and dried with excess anhydrous MgSO_4_. After CH_2_Cl_2_ was removed with a rotary evaporator, the concentrated solution was precipitated with 200 ml of cold ether. The precipitate was dried in a vacuum oven at 40°C to obtain polyethylene glycol-p-toluenesulfonate.

Then, 24 g of polyethylene glycol-p-toluenesulfonate was dissolved in 60 ml of DMF solution. After this, p-hydroxybenzaldehyde (1.98 g, 16.24 mmol) and K_2_CO_3_ (2.24 g, 16.24 mmol) were added to the mixture and stirred for 3 days at 80°C. After cooling to room temperature, 50 ml of deionized water was added to the mixture and then extracted with CH_2_Cl_2_ and precipitated with cold ether. After drying in a vacuum drying oven at 40°C, the OHC-PEG-CHO was obtained.

### 2.4 Synthesis of MSN-PEG

Twenty milligrams of MSN-NH_2_ synthesized in Section 2.2 was dispersed in water, and OHC-PEG-CHO (20 mg) was added and stirred for 2 h. The final product MSN-PEG was obtained by centrifuging at 12,000 rpm for 10 min, and the precipitate was redispersed in deionized water and stored at 4°C.

### 2.5 Preparation of BP

BP was prepared by peeling off BP nanocrystals according to the literature (Aksoy et al., 2020; Zhang et al., 2021). Twenty-five milligrams of BP powder was added to 50 ml of deionized water from which dissolved oxygen had been removed to reduce the oxidation of BP. Then, the mixture was pulverized ultrasonically for 12 h (900 W, SCIENTZ, Ningbo, China). After the color of the BP solution turned dark brown, it was centrifuged at 1,500 rpm for 12 min to remove unstripped BP, and the supernatant was collected and further centrifuged. The supernatant was further centrifuged at 7,800 rpm for 20 min to obtain the BP nanosheets.

### 2.6 Preparation of MSN-PEG@AS/BP Nanoparticles

Five milligrams of AS and 20 mg of MSN-NH_2_ were dissolved in 10 ml of methanol accompanied with magnetic stirring for 24 h. Then, the mixture was centrifuged at 12,000 rpm for 30 min and washed three times with deionized water. The supernatant was collected to calculate the drug loading amount. The resulting precipitate MSN-NH_2_@AS was redispersed in water, and 1 mg of BP was added with stirring. After stirring for 30 min, 20 mg of OHC-PEG-CHO was added and stirred for another 2 h. The final product MSN-PEG@AS/BP was obtained by centrifugation and redispersed in deionized water for storage at 4°C.

### 2.7 Characterization of MSN-PEG@AS/BP

The chemical structures of PEG and OHC-PEG-CHO were confirmed by Fourier transform infrared (FT-IR) spectroscopy (Thermo Electron Scientific Instruments Corp., Fitchburg, WI, United States) and high-resolution proton nuclear magnetic resonance (^1^H NMR, Burke, Germany). Morphology of nanoparticles and nanosheets was observed by a Zeiss EM902 transmission electron microscope (TEM; Carl Zeiss, Germany). Dispersion of nanomaterials was dropped on the copper mesh and dried under room temperature before observation. The size distribution of nanomaterials was analyzed using dynamic laser scatterometer (DLS, Malvern, Zeta Sizer Nano ZS). Concentration of nanomaterials used for DLS testing was 1 mg/ml.

### 2.8 Photothermal Performance Evaluation

The photothermal performance of BP was explored from laser power density and concentration of BP. BP (50 μg/ml, 1 ml) was dispersed in water and irradiated with 808 nm near-infrared (NIR) laser with different laser powers (0.5, 1, 1.5, and 2 W/cm^2^) for 5 min at room temperature and temperature was recorded every 10 s. The photothermal effects of BP at various concentrations (20, 30, 40, and 50 μg/ml) were measured following a similar procedure with a laser power density of 1.5 W/cm^2^. To study the photothermal stability of BP, 1 ml of BP solution (40 μg/ml) was repeatedly irradiated with 808 nm NIR laser (2 W/cm^2^, 5 min) five times.

### 2.9 Material Stability Evaluation

MSN-PEG@AS was dispersed in the PBS buffer (pH = 7.4), and the particle size distribution was measured daily using DLS for 5 days.

### 2.10 Drug Loading and Release Assay

The concentration of AS was measured by a high-performance liquid chromatograph (HPLC) at 205 nm (mobile phase: methanol; 0.8 ml/min) (Szabo et al., 1994). The standard curve of AS is shown in Supplementary Figure S1. The supernatant collected in Section 2.6 was filtered with a 0.22-μm filter and re-dissolved in methanol. The concentration of AS in the supernatant was determined by the standard curve. Drug loading amount and encapsulation efficiency of MSN were calculated by Eqs 1, 2 (Pham et al., 2021).

$$Drug\;loading\;amount\left(\%\right)=W_{1}/W_{0}\times 100\%$$ (1)

$$Encapsulation\;efficiency\left(\%\right)=W_{1}/W_{2}\times 100\%$$ (2)

where *W*_0_ is the total mass of MSN, *W*_1_ is the mass of AS loaded in MSN, and *W*_2_ is the feed amount of AS for drug loading.

MSN-PEG@AS (0.5 mg) was placed in a dialysis bag, and shaken in 2 ml of PBS (pH = 7.4) at 37°C. Release solution (0.2 ml) was taken out, respectively, and the same amount of PBS buffer was added at the same time at different time points (1, 2, 4, 6, 8, 12, 24, 36, 48, and 96 h). The AS concentration was detected by HPLC, and the cumulative release percentage of AS was calculated as reported (Zhang et al., 2017; Chen et al., 2021).

### 2.11 *In Vitro* Antibacterial Activity Evaluation

The antibacterial properties of the nanomaterials were evaluated with Gram-positive *Staphylococcus aureus* (*S. aureus*) and Gram-negative *Escherichia coli* (*E. coli*). Two hundred microliters of nanomaterials suspension (100 μg/ml) was co-incubated with 100 μl of bacteria suspension (1 × 10^6^ CFU/ml), and the MSN-PEG/BP + NIR group was exposed to NIR (2 W/cm^2^) laser for 5 min. After that, 1.7 ml of LB medium was added and incubated at 37°C for 4 h. The diluted bacterial suspension (100 µl) was inoculated on LB agar plates and cultured at 37°C for 24 h. Number of colonies was counted and the antibacterial ratio was calculated by Eq. 3.

$$Antibacterial\;ratio\left(\%\right)=\left(N_{control}-N_{sample}\right)/N_{control}\times 100\%$$ (3)

where *N*_control_ is the average number of colonies in the control sample and *N*_sample_ is the average bacterial colony in the nanomaterials-treated sample.

### 2.12 Cytotoxicity and Live/Dead Staining of L929

L929 in the logarithmic growth phase were used for cytotoxicity assay. Firstly, L929 cells were seeded on a 96-well plate at a density of 1 × 10^4^/well and incubated in a 5% carbon dioxide incubator at 37°C for 24 h. Then, the culture medium was refreshed with culture medium containing 100 μg/ml of nanomaterials and co-incubated for another 24 h. Cell viability was detected using CCK-8 assay per the manufacturer’s instruction, and the optical density at 450 nm was read by a microplate reader. L929 cells co-cultured with nanomaterials in 48 well-plates were further stained by a Live/Dead staining kit (Thermal Fisher, United States). Dead cells were stained with red fluorescence, while live cells were stained with green fluorescence.

### 2.13 *In Vitro* Tube Formation of MSN-PEG@AS/BP

Matrigel (Corning, United States) diluted with serum-free medium at a ratio of 1:1 was added in 48 well-plates and then placed at 37°C to form a gel. HUVECs at a cell density of 10,000 per well were added to the well plate. After the cells adhere to the Matrigel, each group of culture media containing nanomaterials was added separately and incubated at 37°C. Images of HUVEC were taken by an inverted fluorescence microscope at selected time points.

### 2.14 *In Vivo* Infected Full-Thickness Skin Defect Model

Animals were treated in accordance with protocols evaluated and approved by the Ethics Committee of Zhuhai Hospital of Guangdong Provincial Hospital of Chinese Medicine. SD rats (200 g) were randomly divided into five groups, namely, Control (treated with PBS), MSN-PEG@AS, MSN-PEG/BP + NIR, MSN-PEG@AS/BP, or MSN-PEG@AS/BP + NIR. The rats were weighed before the operation and anesthetized with 3% sodium pentobarbital (45–60 mg/kg) intraperitoneally. Hair on the back of rats was shaved and disinfected with iodophor. Full thickness skin on the back was completely removed with tissue scissors to create a 12-mm round defect. The wound was then infected with a mixture of 40 μl of *E. coli* (1 × 10^8^ CFU/ml) and *S. aureus* (1 × 10^8^ CFU/ml) suspension for 1 day before treatment. One hundred microliters of nano-spray (100 μg/ml) was used on the defect every 2 days. The MSN-PEG@AS/BP + NIR group was exposed to NIR laser (2 W/cm^2^) for 5 min after each treatment. Wound area was photographed by a digital camera, and the wound area was evaluated using IPP 6.0 software.

### 2.15 *In Vivo* Antibacterial Performance Analysis

Bacterial number in wounds was evaluated on day 3. Tissue was harvested and homogenate in normal saline, and then 100 μl of diluted homogenate was spread on the Gram-negative bacteria selection medium and mannitol salt agar medium plates, separately. The culture plate was incubated at 37°C for 24 h for colony counting.

### 2.16 Histological and Immunofluorescence Analysis

Skin harvested at 3, 7, 10, and 14 days was fixed in 4% paraformaldehyde for more than 24 h and embedded in paraffin. Tissues were sliced into 4-μm-thick sections and stained according to the manufacturer’s instruction. H&E and Masson staining were used to observe the tissue repair at days 3, 7, 10 and 14. Immunofluorescence staining of TNF-α, TGF-β-1, and IL-10 was used to assess the inflammation level of tissue at days 3 and 7. Immunofluorescence staining of CD31 and α-SMA were used to assess the angiogenesis of tissue at day 7.

### 2.17 Statistical Analysis

Each sample was tested three times in parallel, and the results were expressed in terms of average and standard deviation. The significance was analyzed using GraphPad Prism 7.0. One-way analysis of variance (ANOVA) was performed to evaluate the significance of the experimental data. The statistical significance was *p < 0.05, **p < 0.01, and ***p < 0.001.

## 3 Results and Discussion

### 3.1 Synthesis and Characterization of MSN-PEG@AS/BP

**FIGURE 1:**
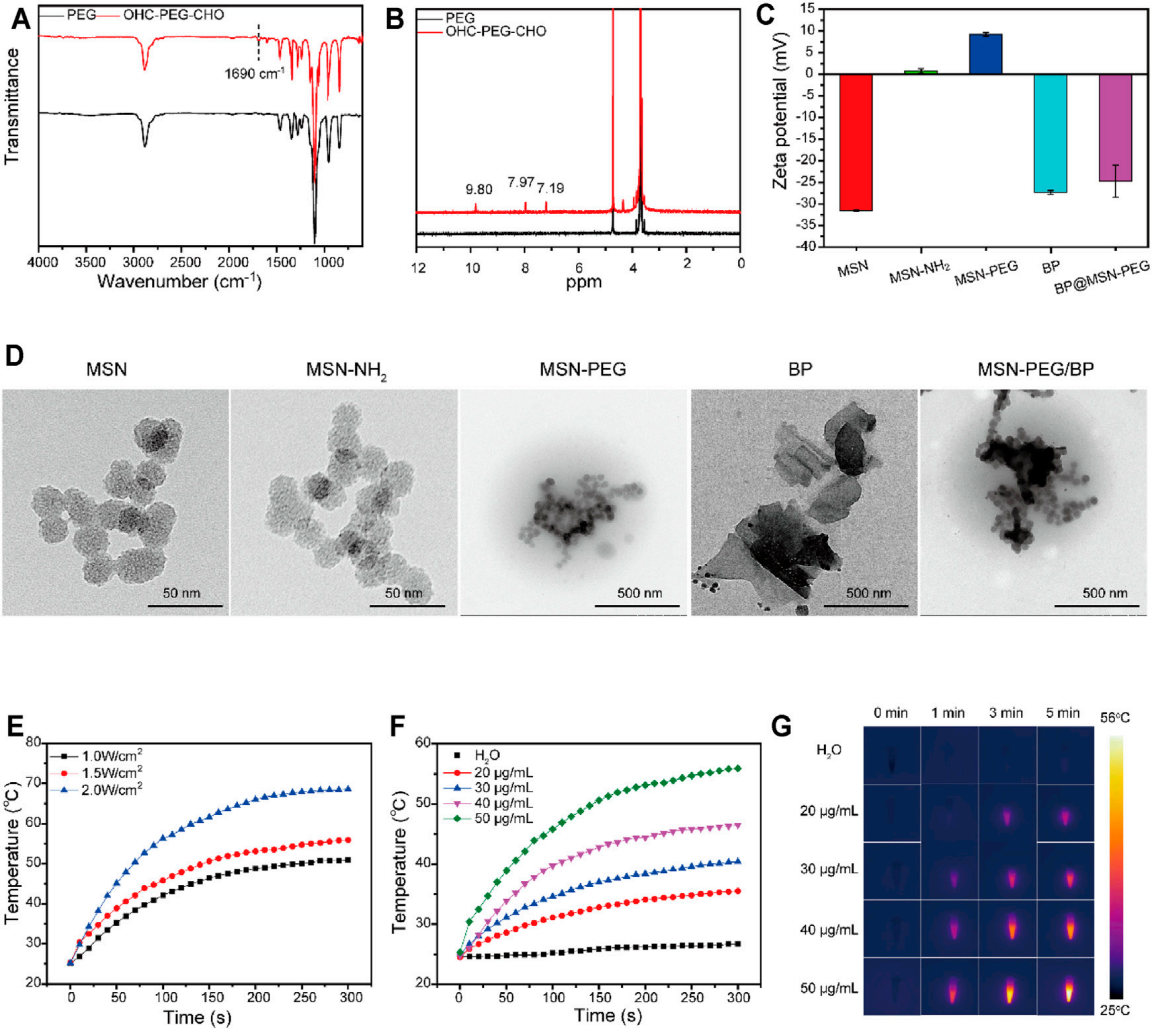
Characterizations of AS@MSN-PEG/BP. **(A)** FTIR spectra of PEG and OHC-PEG-CHO. **(B)** ^1^H NMR spectra of PEG and OHC-PEG-CHO. **(C)** Zeta potential of MSN, MSN-NH2, MSN-PEG, BP, and MSN-PEG/BP. **(D)** TEM image of MSN, MSN-NH2, MSN-PEG, BP, and MSN-PEG/BP. **(E)** Photothermal temperature increase of BP solution (50 μg/ml) under different NIR light irradiation (0.5, 1, 1.5, and 2 W/cm^2^). **(F)** Photothermal temperature increase of DI water and BP solution with different concentrations (20, 30, 40 and 50 μg/ml). **(G)** Photothermal imaging of DI water and BP solution with different concentrations (20, 30, 40, and 50 μg/ml) under NIR light irradiation (1.5 W/cm^2^, 0–5 min).

To prepare the thermally responsive PEG shell for MSN, OHC-PEG-CHO was firstly synthesized as PEG was modified with benzaldehyde groups (Deng et al., 2010). The FT-IR spectra of OHC-PEG-CHO showed the absorption peak at 1,690 cm^−1^ corresponding to the C=O stretching vibration absorption peak (Figure 1A). The ^1^H NMR spectra of PEG and OHC-PEG-CHO showed that the absorption peak at the chemical shift ppm = 9.80 corresponded to the hydrogen on the aldehyde group. Both FT-IR and ^1^H NMR results confirmed the successful modification (Figure 1B).

Aminated MSN was then derived to form MSN-PEG nanoparticles through Schiff base reaction. The zeta-potentials of MSN, MSN-NH_2_, and MSN-PEG (Figure 1C) were −31.5 ± 0.2, 0.794 ± 0.47, and 9.25 ± 0.48 mV, respectively. Obviously, when MSN was modified by amination, the surface charge changed to positive (Aquib et al., 2021). The change of surface charge of MSN was attributed to the appearance of amino groups in MSN (Yang et al., 2017). Compared with MSN-NH_2_, the surface charge of MSN-PEG had a slight increase, perhaps due to the large number of unreacted amino groups on the surface of MSN nanoparticles (von Baeckmann et al., 2021). As shown in Supplementary Figure S2, DLS analysis showed that the particle size of MSN, MSN-NH_2_, and MSN-PEG in PBS solution was approximately 200–260 nm. In addition, the TEM images of MSN and MSN-NH_2_ showed that both MSN and MSN-NH_2_ were approximately spherical porous nanoparticles (Figure 1D), with a particle size of about 20–30 nm. In the TEM image of MSN-PEG, the nanospheres showed an inconspicuous porous structure, and the color deepened, indicating that the PEG shell was successfully modified on the surface of the MSN. The coating of PEG to MSN was due to the Schiff base reaction between MSN-NH_2_ and OHC-PEG-CHO.

The BP nanosheets were peeled ultrasonically from the BP crystals (Ouyang et al., 2018). Figure 1D shows that the BP nanosheets presented a layer structure with a size of 200–600 nm. BP has a negative zeta potential of −27.3 ± 0.451 mV (Figure 1C) (Kong et al., 2020), which can be used for coating with MSN-PEG due to its electrostatic effect. TEM image of MSN-PEG/BP showed that MSN-PEG nanoparticles were successfully deposed on the surface of BP nanosheets (Figure 1D).

Stability of MSN-PEG@AS was also detected. Supplementary Figure S3 showed a particle size distribution of MSN-PEG@AS in PBS buffer within 5 days. The size distribution was increased from 400 nm to about 800 nm in the first 4 days and finally stabilized, owing to the electrostatic effect between MSN-PEG@AS and BP. Finally, the complex showed a stable diameter, indicating that the MSN-PEG@AS/BP have formed a stable complex.

### 3.2 *In Vitro* Photothermal Performance of BP Nanosheets

Photothermal capability of BP is one of the most interesting characteristics for researchers in the field of biomedical engineering, that BP can be used as a new antibacterial agent. The photothermal performance of BP (50 μg/ml) under 808-nm NIR laser irradiation with different power densities is displayed in Figure 1E. With the laser power densities increased (1.0–2.0 W/cm^2^), the temperature of BP increased rapidly and the maximum temperature also increased from 50.9°C to 68.6°C.

Temperature changes of BP solution with different concentrations (20–50 μg/ml) at fixed power density (1.5 W/cm^2^) were also recorded (Figures 1F,G). When the 808-nm NIR laser irradiated for 5 min, the temperature of the BP solution with a concentration of 50 μg/ml reached 55.9°C and increased by 30.6°C. In comparison, the temperature of 40, 30, and 20 μg/ml BP solutions increased by 21.8, 15.4, and 11.0°C, respectively. The temperature of water only increased by 2.1°C in 5 min and showed no obvious change. It could be observed that under the same irradiation time, temperature improved with the increased concentration of the BP solution, which displayed obvious concentration dependence (Shao et al., 2016). Additionally, the temperature of the BP solution increased rapidly in a short time under the irradiation of the NIR laser and gradually slows down until it stabilizes with the increase of irradiation time.

Photothermal stability was also analyzed as shown in Supplementary Figure S4. After three cycles of repeated NIR laser irradiation, the temperature increase of BP was about 25.9°C, and the temperature could still rise sharply in a short period of time without significant difference compared to the first cycle, indicating its excellent photothermal stability as reported (Liu et al., 2020).

### 3.3 *In Vitro* Drug Loading and Release Behavior

To verify the drug loading and release ability of the MSN-PEG@ AS, the AS loading and release was investigated. Drug loading and encapsulation efficiency of AS was 8.12 and 70.7%, respectively, according to HPLC determination. Supplementary Figure S5 shows the *in vitro* release curve of MSN-PEG@AS. AS showed a quick release in the first 8 h, and nearly 40% released. Most of the AS molecules entrapped in the MSN were quickly released into the aqueous medium. The initial sharp burst was due to the rapid leaching of AS from the hole. The release gradually slowed down in the later period, because the concentration of AS in the aqueous medium gradually increased, and the release balance was gradually reached (Xue and Shi, 2004). The release balance showed that 91.32 ± 2.75% of AS was released at 96 h. That means, AS can take effect within 4 days, confirming the sustained-release effect of MSN and providing support for reducing the frequency of administration.

### 3.4 *In Vitro* Antibacterial Activity Evaluation

**FIGURE 2:**
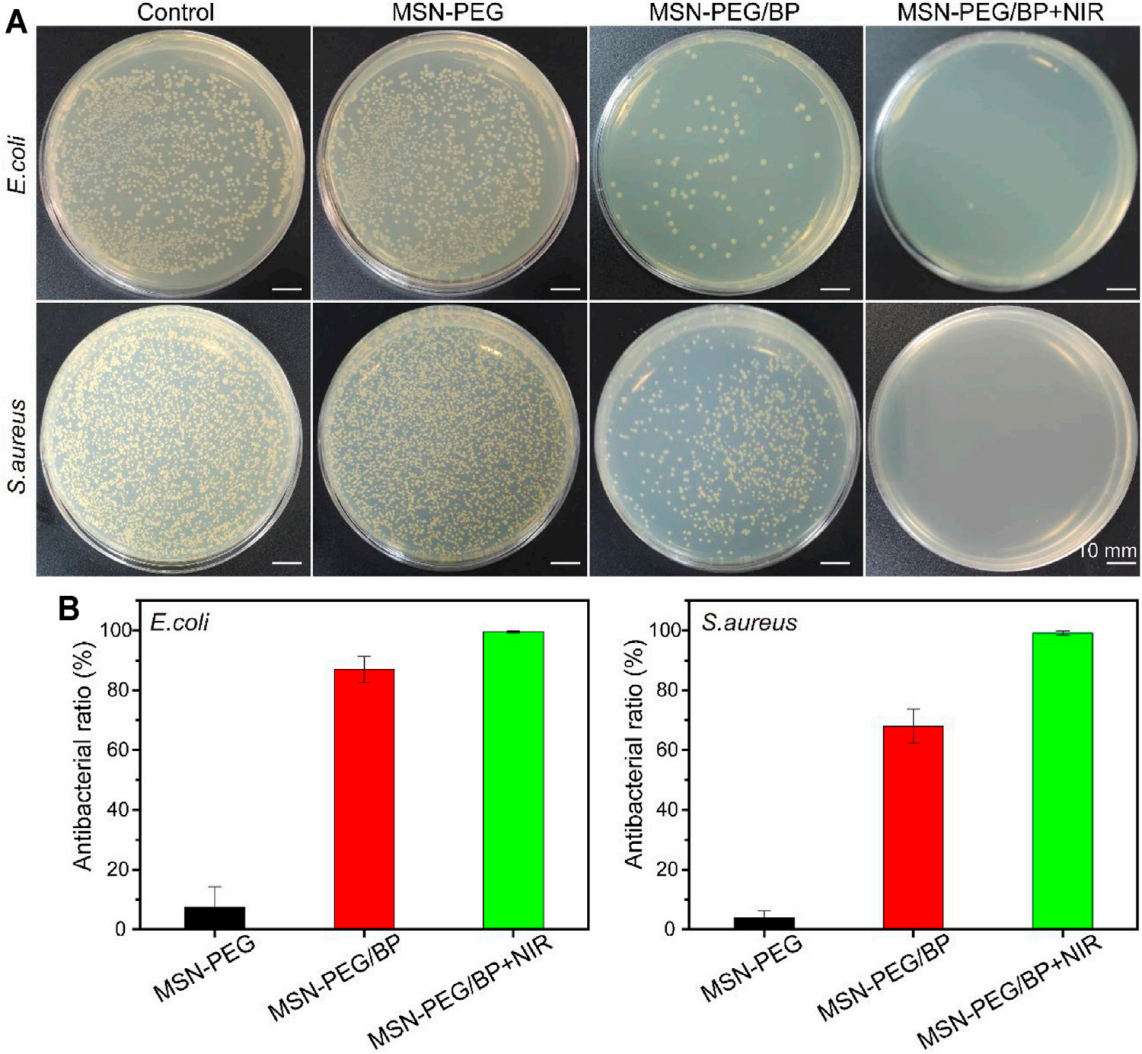
**(A)** Agar Petri dish images of *E. coli* and *S. aureus* bacterial colonies after the treatments and **(B)** their corresponding survival rates *in vitro*: (1) Control, (2) MSN-PEG, (3) MSN-PEG/BP, and (4) MSN-PEG/BP + NIR.

The antibacterial activity against *E. coli* and *S. aureus* was evaluated. As shown in Figure 2A, the MSN-PEG group caused the death of only a very small number of *E. coli* and *S. aureus*, which was equivalent to that of the control group. In contrast, the number of *E. coli* and *S. aureus* treated by MSN-PEG/BP without NIR group was significantly reduced, indicating that BP itself can cause certain bacterial toxicity in the absence of NIR. Xiong et al. have clearly demonstrated that the sharp edges of BP nanosheets can cause bacterial membrane damage (Xiong et al., 2018). The bacteria treated with MSN-PEG/BP were almost completely killed under NIR irradiation, which is attributed to the toxicity of BP to bacteria and the high temperature under NIR laser irradiation. Figure 2B quantitatively showed the outstanding antibacterial effect of MSN-PEG/BP, with antibacterial rates of 99.58% (*E. coli*) and 99.13% (*S. aureus*), respectively. Therefore, MSN-PEG/BP has a great potential for antibacterial applications.

### 3.5 Evaluation of Biocompatible and Angiogenic Potential

**FIGURE 3:**
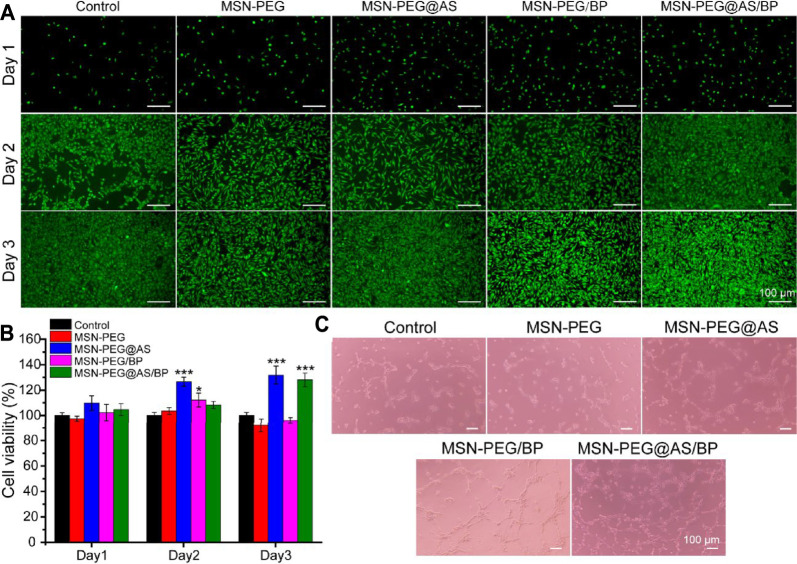
**(A)** Live/dead staining and **(B)** cytotoxicity assay of L929 cells at different time points. **(C)** The angiogenic potential of HUVEC treated with different nanomaterials (100 μg/ml).

Cell cytotoxicity against L929 cells was conducted to evaluate the biocompatibility of the materials. Figure 3A shows that the live/dead staining of L929 co-incubated with nanomaterials. Green fluorescence represents the live cells, while red fluorescence indicates the dead cells. The cells showed a good spread morphology, and the number gradually increased with the extension of the culture time. All cells co-cultured with nanomaterials did not show red fluorescence, which means nanomaterials based on MSN-PEG and BP has no obvious cytotoxicity to L929.

The cell viability of L929 cells treated with different nanomaterials was quantitively analyzed using the CCK-8 kit and is displayed in Figure 3B. All groups showed cell viability higher than 97.5% after culturing for 24 h, indicating no cytotoxicity according to ISO 10993-5:2009. Nanomaterials containing AS all promoted the proliferation of L929, suggesting that AS can promote the proliferation of fibroblasts as described in the literature (Chen et al., 2012).

Furthermore, the pro-angiogenic properties of the prepared materials were evaluated using Matrigel by the tube formation assay. Compared with the control group, the effect of tubule formation in the MSN-PEG group was negligible (Figure 3C). On the contrary, the MSN-PEG@AS group and MSN-PEG/BP group have more tubule formation and branch points than the MSN-PEG group, which is probably due to AS and BP affecting the formation of blood vessels (Wang et al., 2013; Qian et al., 2019). In addition, the MSN-PEG@AS/BP group had maximal tubule formation and branch points, indicating the best angiogenesis effect compared to other groups.

### 3.6 *In Vivo* Wound Healing and Antibacterial Ability

**FIGURE 4:**
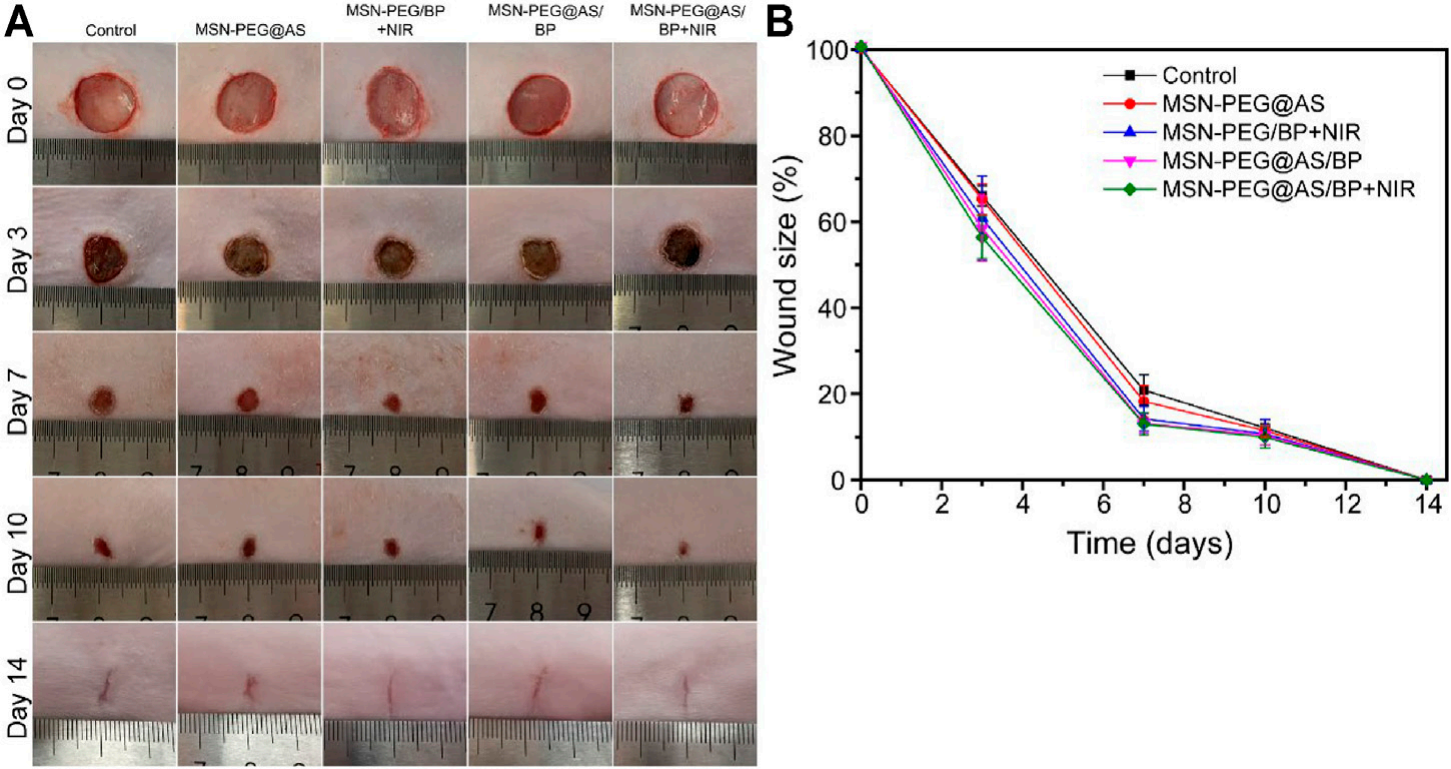
**(A)** Digital image of wound healing at day 0, 3, 7, 10, and 14 with different treatments. **(B)** Quantification of wound closure rate (Control, MSN-PEG@AS, MSN-PEG@BP + NIR, MSN-PEG@AS/BP, and MSN-PEG@AS/BP + NIR).

The *in vivo* application of the MSN-PEG@AS further confirmed the successful treatment of chronic wounds. Healthy SD rats were randomly divided into five groups: Control, MSN-PEG@AS, MSN-PEG/BP + NIR, MSN-PEG@AS/BP, and MSN-PEG@AS/BP + NIR. The images of the wounds were taken by a digital camera for gross observation (Figure 4A). The relative wound area showed the nanomaterials with AS leading to a smaller wound area, which was conspicuously smaller than the control group, indicating that the AS could promote wound healing as reported in the literature (Lee et al., 2018). Moreover, the wound healing in the rats treated with MSN-PEG@AS/BP with or without NIR irradiation occurred faster than in the rats treated without BP at day 3.

More specifically, Figure 4B further confirmed this finding as the wound size of the MSN-PEG@AS/BP with NIR irradiation group was smaller than that of the group without NIR irradiation. At days 7 and 10, MSN-PEG@AS/BP with NIR irradiation-treated wound showed the smallest area, 56.3% and 12.5%, respectively, which revealed that the MSN-PEG@AS/BP with NIR irradiation could be used for the bacteria-infected wound *in vivo* with the assistance of photothermal effects.

### 3.7 *In Vivo* Antibacterial Performance Analysis

**FIGURE 5:**
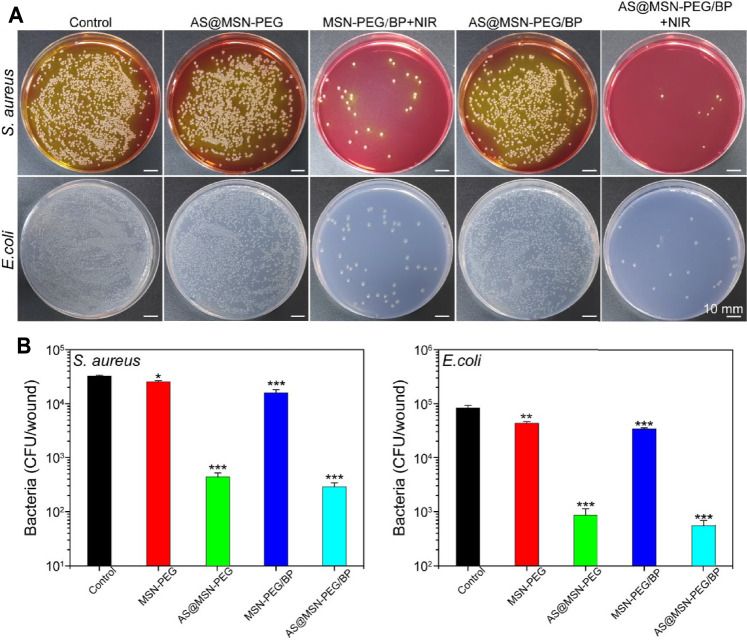
**(A)** Digital images of *E. coli* and *S. aureus* bacterial colony-forming units, obtained from the wound tissue (day 3) under various experimental conditions (Control, MSN-PEG@AS, MSN-PEG@BP + NIR, MSN-PEG@AS/BP, and MSN-PEG@AS/BP + NIR). **(B)** Quantitative results of standard plate counting assay after 24 h (*n* = 3).

The *in vivo* antibacterial performance of the materials was evaluated by the full-thickness skin defect model mentioned above. We collected the infected skin tissue in the wounds and tested the bacterial viability by a colony counting assay to quantitatively evaluate the growth of bacteria in the wound on day 3. The growth of bacteria in the wounds was observed in Figure 5A. The colony numbers of *E. coli* and *S. aureus* in the MSN-PEG/BP + NIR group and MSN-PEG@AS/BP + NIR group were much lower than those of other groups. Moreover, nearly 30,000–15,000 CFU/wound of *E. coli* and 3000-8000 CFU/wound of *S. aureus* were still alive in the wound site in the control group, MSN-PEG@AS group, and MSN-PEG@AS/BP without NIR irradiation group (Figure 5B). In contrast, the number of *E. coli* and *S. aureus* colonies in the group with NIR irradiation was much lower than that in the group without NIR irradiation, about 500-800 CFU/wound and 200-400 CFU/wound, respectively. The colony count statistics of wounds in different groups demonstrated that the good antibacterial effect *in vivo* was attribute to the high temperature caused by BP with NIR irradiation.

### 3.8 Wound Histological Analysis

**FIGURE 6:**
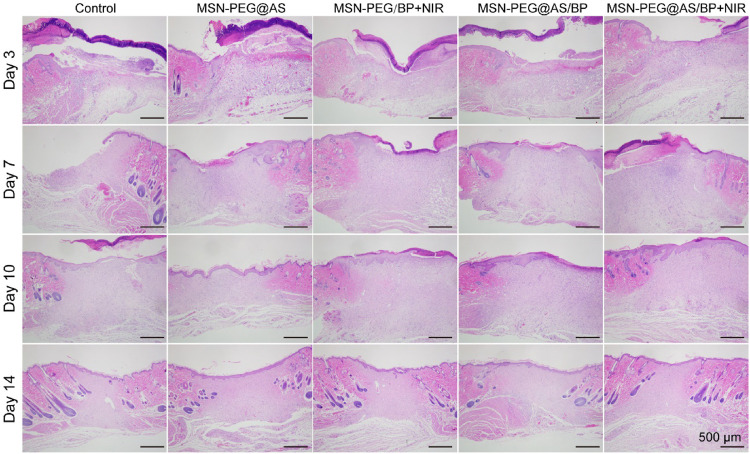
H&E staining of wound sections at days 0, 3, 7, 10, and 14 with different treatments (Control, MSN-PEG@AS, MSN-PEG@BP + NIR, MSN-PEG@AS/BP, and MSN-PEG@AS/BP + NIR).

The wound healing quality was further evaluated by H&E and Masson staining. After 3 days of treatment, the granulation tissue was observed in the wounds. Additionally, compared with the control group, the thickness of the granulation tissue in the nano-spray-treated groups was significantly higher at days 3, 7, and 10 (Figure 6). In the early stage of wound healing, the secreted pro-inflammatory cytokines and growth factors promote the proliferation of fibroblasts, the differentiation of immune cells, and the deposition of ECM to form the new granulation tissue. At day 7, the intact neoepidermis layer was formed in the wound sites of the MSN-PEG/BP + NIR group, the MSN-PEG@AS/BP with NIR group, and the MSN-PEG@AS/BP without NIR irradiation group compared with other groups. In contrast, the little granulation tissue and the thin neoepidermis layer were barely observed in the wounds treated with PBS and MSN-PEG@AS. In fact, the epithelium of the MSN-PEG@AS/BP + NIR group was smoother and more regulated than that of the MSN-PEG@AS/BP group, and follicles began to develop around the wound.

**FIGURE 7:**
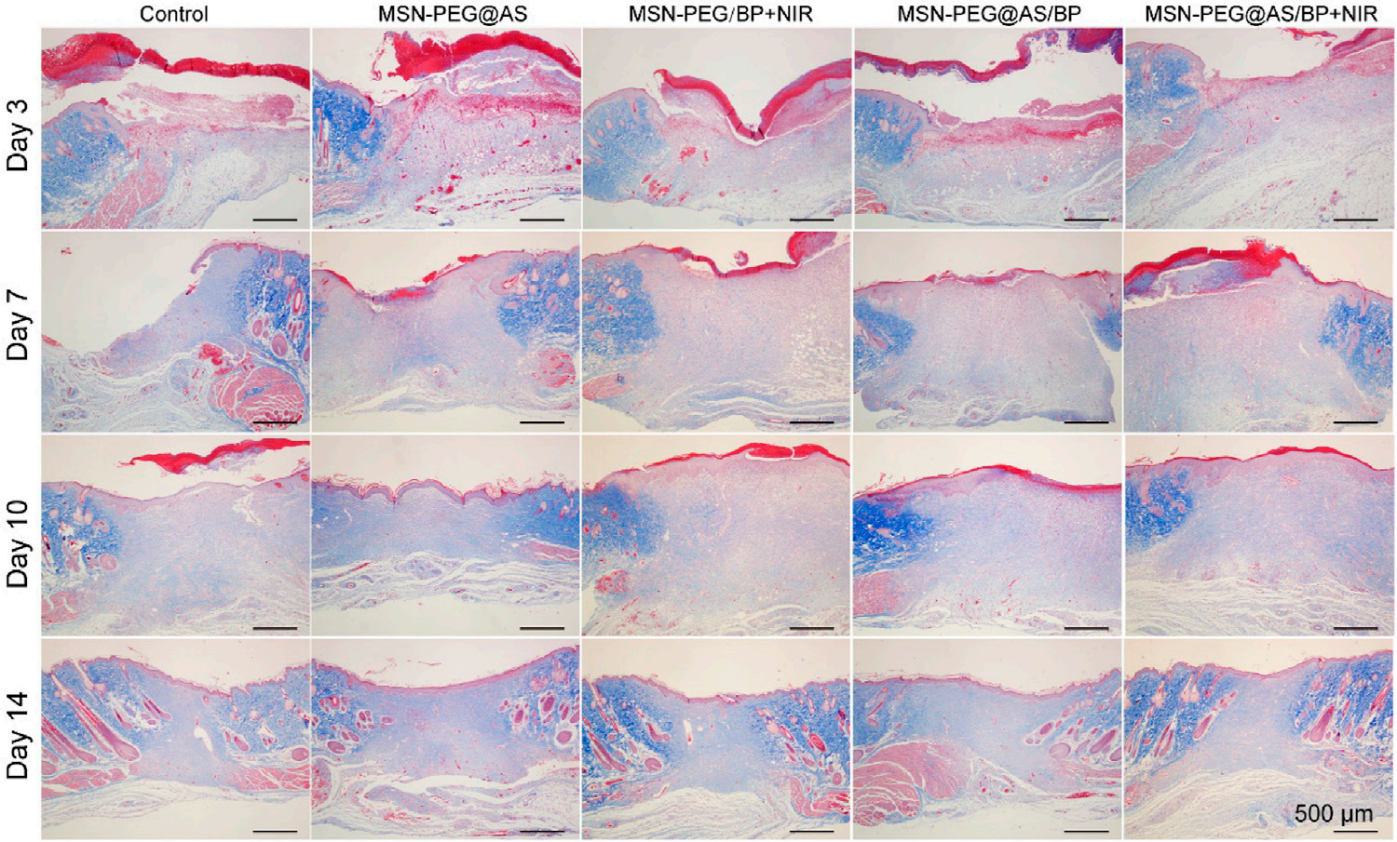
Masson staining of wound sections at days 0, 3, 7, 10, and 14 with different treatments (Control, MSN-PEG@AS, MSN-PEG@BP + NIR, MSN-PEG@AS/BP, and MSN-PEG@AS/BP + NIR).

Besides, Masson staining was applied to different groups to reflect the deposition of collagen. Similarly, there was a larger collagen deposition area at wound with dense collagen fibers in the MSN-PEG@AS/BP + NIR group (Figure 7). As one of the main components of the dermis, the deposition of collagen plays an important role in wound healing. The increased collagen content in the wound demonstrated a faster wound regeneration rate promoted by the MSN-PEG@AS/BP with NIR.

### 3.9 *In Vivo* Anti-Inflammatory and Angiogenesis

**FIGURE 8:**
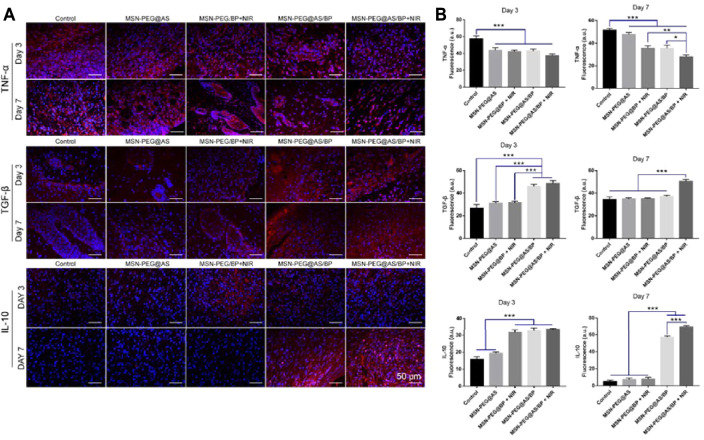
**(A)** Immunofluorescence staining of TNF-α, TGF-β, and IL-10 in the wounds at Day 3 and Day 7. **(B)** Quantitative analysis of Immunofluorescence staining of TNF-α, TGF-β, and IL-10 in the wounds at Day 3 and Day 7.

In order to further explore the changes in the wound microenvironment after treatment, we analyzed the skin samples by immunofluorescence staining. Tumor necrosis factor-alpha (TNF-α) secreted by proinflammatory macrophages has negative effects on the wound microenvironment (Ashcroft et al., 2012). Figure 8A shows that the positive expression of TNF-α in each group of wounds on the seventh day was lower than that on the third day. The expression of TNF-α in wounds treated with MSN-PEG@AS/BP with NIR was significantly lower than that of other groups. The low expression of TNF-α could lead to less ECM proteolysis, which is consistent with the H&E staining results mentioned above. Figure 8B is the fluorescence quantitative analysis corresponding to Figure 8A, and the above analysis results can be observed from it more intuitively.

Moreover, we also detected the positive expression of TGF-β and IL-10 in the samples. The literature has reported that TGF-β triggers fibroblasts and myofibroblasts to produce new ECM, which plays a central role in fibrinogenesis (Schiller et al., 2004). Besides, IL-10 is an anti-inflammatory cytokine that could induce polarization of macrophages (Ferrante and Joseph Leibovich, 2012). Compared to day 3, the expression of TGF-β and IL-10 at the wound site was significantly upregulated on day 7, and the expression in the MSN-PEG@AS/BP with NIR group was the most. Briefly, MSN-PEG@AS/BP can regulate the secretion of anti-inflammatory cytokines and inhibit the expression of TNF-α to change the wound microenvironment from a pro-inflammatory into a pro-resolution state and promote matrix regeneration and vascular remodeling.

**FIGURE 9:**
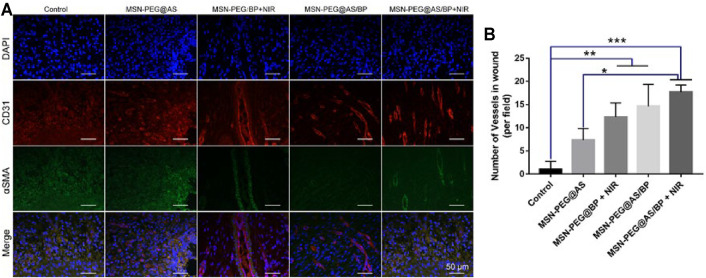
**(A)** Immunofluorescence staining of DAPI, CD31, and α-SMA in the wounds at Day 7. **(B)** Quantitative analysis of number of blood vessels.

In addition, we detected the expression of CD31 and α-SMA in the samples on day 7 to assess the formation of blood vessels. Angiogenesis is essential for the healing of chronic wounds. Compared with other groups, the expression of CD31 and α-SMA was almost invisible in the wound treated by the control group and MSN-PEG@AS group (Figure 9A). However, the co-localizations of CD31 and α-SMA positive staining treated by the MSN-PEG@AS/BP with NIR group were significantly more than the other groups, which is consistent with the less expression of TNF-α mentioned above. A prior study reported that the reduction of TNF-α promotes the expression of α-MSA in human dermal fibroblasts (Goldberg et al., 2007). Figure 9B shows the statistics of blood vessel density at the corresponding wound site. The results also demonstrated that the addition of MSN-PEG@AS/BP with NIR had a noteworthy effect on promoting angiogenesis during the wound healing process.

## 4 Conclusion

In summary, we reported novel photothermal 2D nanosheets combined with AS (MSN-PEG@AS/BP) that have antibacterial properties and that promote angiogenesis to treat infected wounds. The MSN-loaded AS exhibits excellent angiogenesis and anti-inflammatory properties, which showed excellent cytocompatibility and played a powerful regulation effect for inflammatory response and enhanced the formation of blood vessels *in vivo*. In addition, the 2D nanosheet BP showed outstanding photothermal property, favorable photothermal stability, and excellent antibacterial effect with antibacterial rates of 99.58% (*E. coli*) and 99.13% (*S. aureus*). Therefore, as an antibacterial and biocompatibility nano-spray, MSN-PEG@AS/BP may be an outstanding candidate for infected wound treatment.

## Data Availability

The original contributions presented in the study are included in the article/Supplementary Material, further inquiries can be directed to the corresponding author.
