# Supplementary material for: Photothermal 2D Nanosheets Combined With Astragaloside IV for Antibacterial Properties and Promoting Angiogenesis to Treat Infected Wounds
Source: Front Bioeng Biotechnol. 2022 Feb 9;9:826011. doi: 10.3389/fbioe.2021.826011 (PMC8864217; doi:10.3389/fbioe.2021.826011)
Supplement: Supplementary file 1 [file DataSheet1.docx]

**Supporting Information**

**Photothermal 2D Nanosheets Combined with Astragaloside IV for Antibacterial Properties and Promoting Angiogenesis to Treat Infected Wounds**

Lichang Liu^a^, Wenfeng Wang^a^, Weihong Hong^a^, Yuyan Jin^a^, Lichun Wang^a^, Sujun Liu^a^, Ailin Wang^b^, Xusheng Liu^c^*****

^a^ Department of Nephrology, Zhuhai Hospital of Guangdong Provincial Hospital of Chinese Medicine, Zhuhai, China;

^b^ Second Clinical Medical College, Guangzhou University of Chinese Medicine, Guangzhou, China;

^c^ Department of Nephrology, Guangdong Provincial Hospital of Chinese Medicine, Guangzhou, China

*****Correspondence to: liuxu801 @ 126.com (Xusheng Liu).





**Figure S1.** The standard curve equation of AS drug.

**
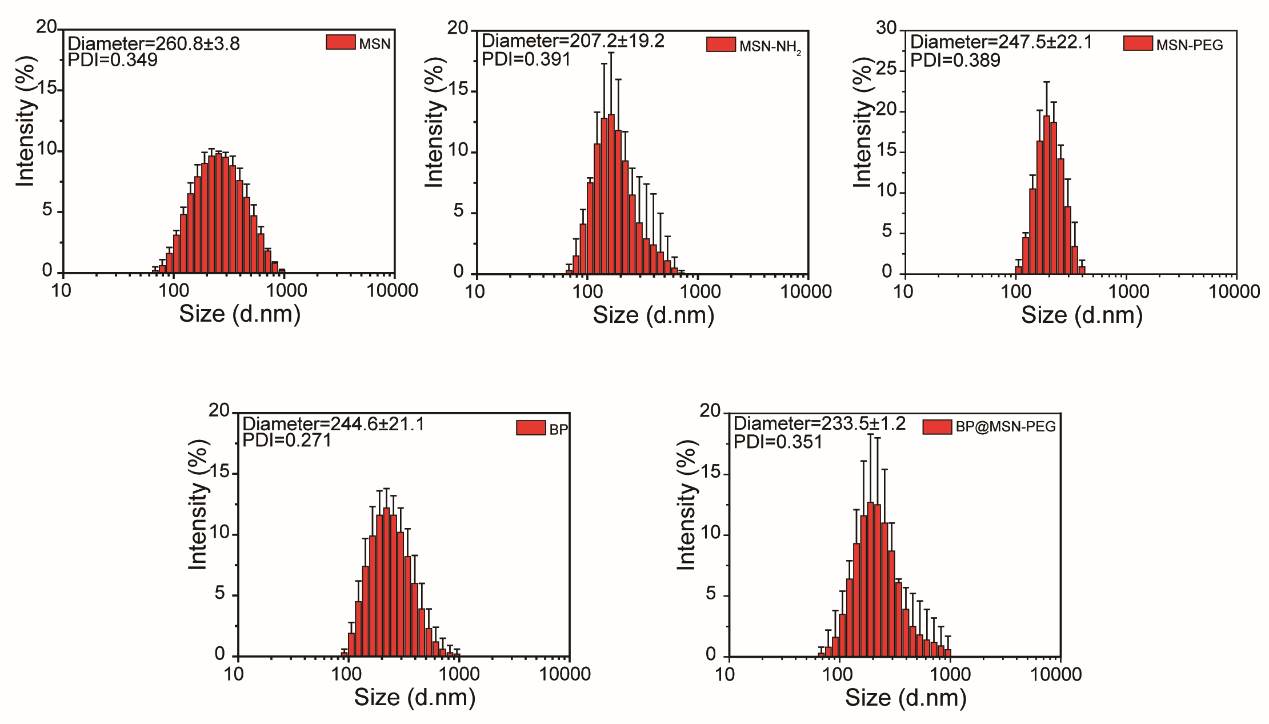
**

**Figure S2.** The particle size of MSN, MSN-NH2 and MSN-PEG in PBS solution.





**Figure S3.** The particle size of MSN-PEG@AS in PBS solution within 5 days.


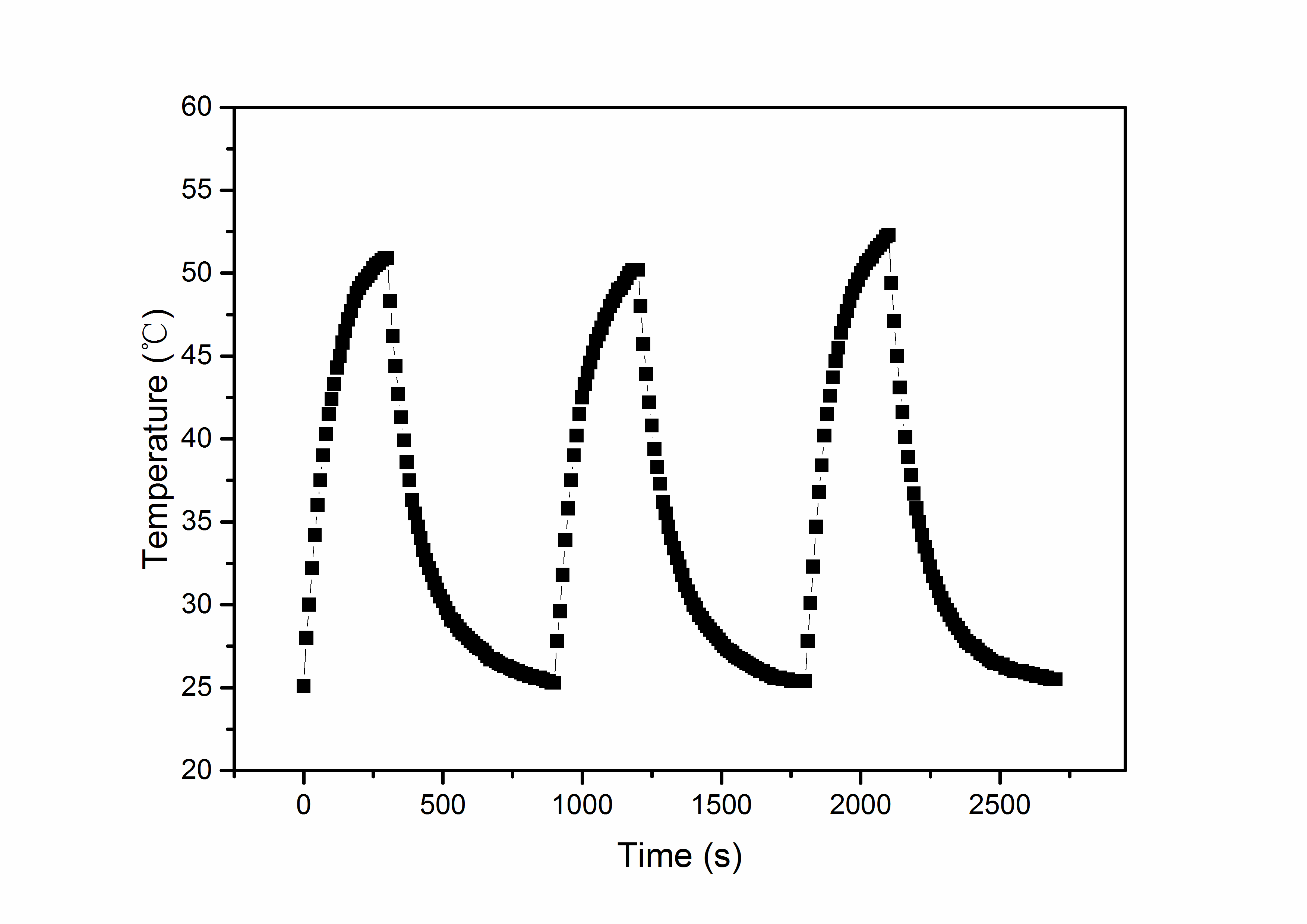


**Figure S4.** Photothermal temperature change of BP solution (40 ug/ml) under 3 cycles of repeated NIR laser irradiation (2 W/cm^2^, 5 min).





**Figure S5.** The release profile of AS drug from MSN-PEG@AS for 7.4 at different time points (1, 2, 4, 6, 8, 12, 24, 36, 48, 96 h).
